# Range‐Wide Assessment of the Tasmanian Devil Gut Microbiome

**DOI:** 10.1002/ece3.71196

**Published:** 2025-05-04

**Authors:** Meadhbh M. Molloy, Elspeth A. McLennan, Samantha Fox, Katherine Belov, Carolyn J. Hogg

**Affiliations:** ^1^ School of Life and Environmental Sciences The University of Sydney Sydney New South Wales Australia; ^2^ Department of Environmental Science and Policy George Mason University Fairfax Virginia USA; ^3^ Save the Tasmanian Devil Program Department of Natural Resources and Environment Hobart Tasmania Australia; ^4^ Toledo Zoo and Aquarium Toledo Ohio USA

**Keywords:** amplicon sequencing, conservation, diet, metabarcoding, microbiome, threatened species

## Abstract

The gut microbiome is an important component of host health and function and is influenced by internal and external factors such as host phylogeny, age, diet, and environment. Monitoring the gut microbiome has become an increasingly important management tool for wild populations of threatened species. The Tasmanian devil ( *Sarcophilus harrisii* ) is the largest extant carnivorous marsupial from the island state of Tasmania, Australia. Devils are currently endangered due to devil facial tumor disease. Previous assessments have shown differences between captive and wild devil gut microbiomes and changes during translocations. However, wild gut microbiome variability across Tasmania and the drivers of these differences are not well understood. We conducted a range‐wide assessment of gut microbiomes at 10 locations across Tasmania, via 16S rRNA sequencing, and tested the influence of diet (12S vertebrate sequencing), location, sex, and cohort. We show that the five most abundant phyla and genera were consistent across all 10 locations. Location, cohort, and sex impacted bacterial richness, but location did not impact diversity. While there were differences in diet across the state, there was no strong evidence of differences between juveniles and adults, nor between males and females. Contrary to our hypothesis, the vertebrate diet explained a small amount of variation in microbial communities. We suspect that other variables, such as environmental factors and immune system development, may have a stronger influence on gut microbiome variability. Dietary components missed by our 12S primer, including invertebrates and plants, may also contribute to these patterns. Adjustments to dietary supplementation are not recommended when preparing devils for translocation to different sites. Future research should prioritize collecting environmental samples for microbial analysis and integrating metabolomics to elucidate functional differences associated with Tasmanian devil gut microbiome variability.

## Graphical abstract

We conducted a comprehensive assessment of gut microbiomes at 10 locations across Tasmania, utilizing molecular methods such as 16S rRNA amplicon sequencing and diet metabarcoding to investigate the influences of diet, location, sex, and age. Contrary to the prevailing notion that diet is a main driver of gut microbiome differences, we found that dietary differences accounted for only a small proportion of microbial variation, suggesting that other ecological and physiological factors play a more pivotal role.

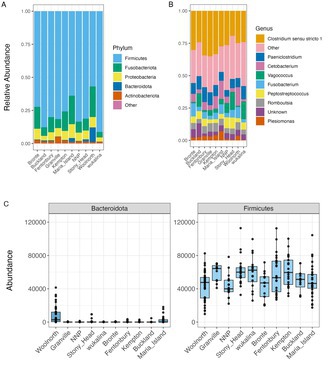

## 1 Introduction

The collection of microbes in the intestines of a host that exist in a dynamic state is known as the gut microbiome (Thursby and Juge 2017). Most microbiome research is driven by human‐focused questions. In humans, the gut microbiome plays an important role in host health by supporting nutrition absorption (Walter and Ley 2011), immune development (Hooper et al. 2012), and defense against pathogens (Spragge et al. 2023). Any change considered to be abnormal to the gut microbiome can cause “dysbiosis”; a general term that refers to the deviation from a normal state that can lead to an increase in pathogenic bacteria and a subsequent increase in disease risks (Cerf‐Bensussan and Gaboriau‐Routhiau 2010). While gut microbiomes are largely explored in the context of human health, recognition that the gut microbiome can inform the management of wild species is rapidly increasing (Redford et al. 2012; Trevelline et al. 2019; Bornbusch et al. 2024).

The gut microbiome can be influenced by a range of factors, including host phylogeny and diet (Ley et al. 2008). Interactions of diet and gut microbiome composition were documented in several wild African ungulate species (Kartzinel et al. 2019), Arunachal macaques ( *Macaca munzala* ; Ghosh et al. 2021), koalas ( *Phascolarctos cinereus* ; Blyton et al. 2023), and Grauer's gorillas ( *Gorilla beringei graueri* , Michel et al. 2023). Geographic location can also influence gut microbiomes, as differences were documented in Australian sea lions ( *Neophoca cinerea* ; Delport et al. 2016) and southern greater gliders ( *Petauroides volans* ; Clough et al. 2023). With threatened species management, a particular focus is the effect of captivity—a necessary part of maintaining insurance populations—on the host microbiome. While there is growing interest in the gut microbiome's role in wildlife health, direct empirical evidence linking dysbiosis to specific diseases in threatened species is still relatively scarce compared to human studies. However, captive populations of threatened species are known to have higher rates of obesity, disease risks when reintroduced to the wild, and lower rates of breeding success, which are factors broadly associated with gut microbiome alterations (Dallas and Warne 2023). Responses to captivity may vary based on taxonomic group. For example, a meta‐analysis of captivity and gut microbiomes across different mammalian groups in captivity found decreased alpha diversity in canids, primates, and equids, increased diversity in rhinoceros, and no effect in bovids, giraffes, anteaters, and aardvarks (McKenzie et al. 2017). As gut microbiome responses to differences in environment are not standard across taxa, it is important to tailor management strategies to the specific needs and responses of each species.

The Tasmanian devil ( *Sarcophilus harrisii* ; or “devil”) is the largest extant marsupial carnivore that is endemic to the island state of Tasmania, Australia. Devils are listed as endangered due to devil facial tumor disease (DFTD), an infectious clonal cancer that has caused declines of over 82% in some wild populations since it was first observed in 1996 (Cunningham et al. 2021). Devils infected with DFTD suffer from large tumors that concentrate around the head, which impedes feeding and breathing and increases susceptibility to secondary infections (McCallum and Jones 2006). In response to the high mortality rate caused by DFTD, the Tasmanian devil insurance population commenced in 2006 as a collaboration between the Save the Tasmanian Devil Program (STDP) and the zoo industry (Hogg et al. 2017). As part of the metapopulation management strategy, zoo‐born devils raised in captivity were translocated to Maria Island off the coast of Tasmania, and their offspring were used as part of the trial Wild Devil Recovery translocation project (Hogg et al. 2019, 2020; Fox and Seddon 2019).

The first study that characterized Tasmanian devil gut microbiomes found differences between captive and wild populations, with captive devils having notably decreased diversity and richness in their microbiomes (Cheng et al. 2015). However, devils adopted a wild‐type microbiome within 6–12 months post‐translocation, with characteristics such as a high Firmicutes: Bacteroidota (F:B) ratio observed in the wild (Chong et al. 2019) commonly seen in other carnivorous mammals (Ley et al. 2008). While this high F:B ratio is associated with potential metabolic disease in humans (Fan and Pedersen 2021; Houtman et al. 2022), it may be a necessity for some carnivorous species that require higher capacity for energy harvest (Heiss and Olofsson 2018). Tasmanian devils are opportunistic carnivores and scavengers with a wide dietary range (Pemberton et al. 2008; McLennan et al. 2022). Diet surveys with scat contents (Jones and Barmuta 1998; Pemberton et al. 2008, Andersen et al. 2017) and video collars (Andersen et al. 2020) have confirmed that devils mainly consume mammals and birds. Previous research has found that devils younger than 12 months of age had “broader group isotope niches” compared to devils that were 12 months and older, suggesting that juvenile devils may have a more diverse diet compared to adults (Bell et al. 2020). Based on the mounting evidence linking diet to gut microbial diversity and composition in other mammalian wildlife (Kartzinel et al. 2019; Blyton et al. 2023; Michel et al. 2023), we expect that Tasmanian devils with different diets are likely to also have differences in their gut microbiomes. Exploring these differences is available through targeted metabarcoding, which is an effective technique for diet analysis as it allows for the broad capture of different taxonomic groups.

Many studies have used metabarcoding for diet assessments to monitor wildlife. For example, Eurasian otters ( *Lutra lutra* ) were found to consume different prey when outside of their reserve (Wang et al. 2022); introduced species were found to be the predominant group in the diet of the Telfair's skink ( *Leiolopisma telfairii* ; Tercel et al. 2022) and understanding diet preference in the hairy‐nosed wombat ( *Lasiorhinus krefftii* ) allowed for the identification of future relocation sites (Casey et al. 2023). The main diet taxa consumed by the endangered black‐faced spoonbill ( *Platalea minor* ) were identified, helping to understand what food resources are important for a managed population to have in a semi‐artificial habitat (Huang et al. 2021). Metabarcoding was also used to show how different sampling techniques can impact the accuracy of diet analyses in cheetahs ( *Acinonyx jubatus* ; Thuo et al. 2019) and was found to be more effective than stable isotope analysis for platypus ( *Ornithorhynchus anatinus* ) diet analyses (Hawke et al. 2022). Through metabarcoding, the impact of carnivore translocations on a new habitat was better understood for Tasmanian devils introduced to Maria Island, Tasmania, where devils were found to consume invasive species such as domestic cats ( *Felis catus* ), and vulnerable species including short‐tailed shearwaters ( *Puffinus tenuirostris* ) and little penguins ( *Eudyptula minor* ; McLennan et al. 2022).

In this study, we aimed to characterize the Tasmanian devil gut microbiome across the entire state of Tasmania by exploring differences between diet (using 12S metabarcoding), location, sex, and age cohort. Specifically, our objectives were to (1) characterize the spatial variation in microbiome and diet within each location (alpha diversity) and between the different locations (beta diversity), (2) explore the amplicon sequence variants (ASVs) influencing spatial variation in the gut microbiome through differential abundance analyses, and (3) explore correlations between gut microbiome profiles and diet.

## 2 Materials and Methods

### 2.1 Sample Collection

**FIGURE 1:**
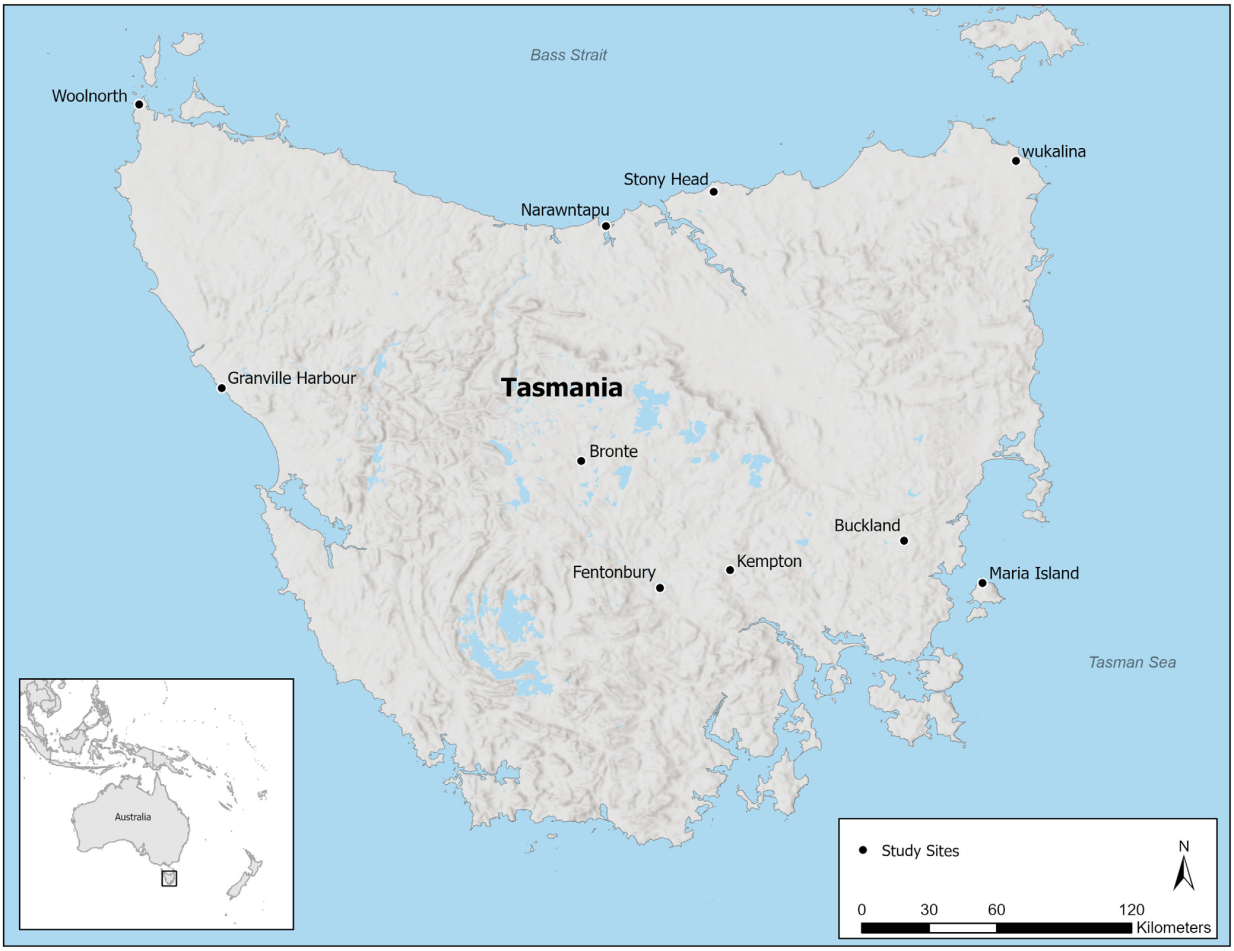
Map featuring the 10 sites where Tasmanian devils were trapped during the February to June 2022 trapping season. Fecal samples were collected from the following sites: Maria Island (*n* = 31), wukalina (*n* = 16), Buckland (*n* = 12), Stony Head (*n* = 21), Narawntapu National Park (*n* = 16), Fentonbury (*n* = 23), Bronte (*n* = 13), Kempton (*n* = 21), Granville Harbor (*n* = 11), and Woolnorth (*n* = 35). Included is a map showing Tasmania in relation to mainland Australia, a scale representing kilometers, and a north arrow indicating direction.

Samples were collected between February and July 2022 by Save the Tasmanian Devil Program (STDP) field staff during routine monitoring at 10 locations across Tasmania (Figure 1). Samples were collected from either the PVC pipe trap during nightly trapping or from the handling sack during processing upon the first capture of the devil for any given trapping trip. Host age, sex, individual identifier (microchip), and DFTD score (1–5, with 1 being no confirmed DFTD and 5 being a severe case of DFTD) were recorded for each individual sample. Only animals with a DFTD score of 1 (“no‐DFTD”) were included in this study. This resulted in a total of 199 fecal samples from Maria Island (*n* = 31), wukalina (*n* = 16), Buckland (*n* = 12), Stony Head (*n* = 21), Narawntapu National Park (hereafter “NNP”; *n* = 16), Fentonbury (*n* = 23), Bronte (*n* = 13), Kempton (*n* = 21), Granville Harbour (*n* = 11), and Woolnorth (*n* = 35; Figure 1). All samples were stored at −20°C immediately after field collection. Due to the limited and uneven sample size between different ages, the data were sorted into two different age groups (hereafter, “cohort”), with “juveniles” being devils 1 year of age or younger, and “adults” as devils that are two or more years of age. All sample collection procedures were approved by The University of Sydney's Ethics Committee (Animal Ethics Approval Number: 2022/2243) and conducted in accordance with relevant guidelines and regulations.

DNA was extracted in a dedicated clean laboratory within a biosafety cabinet following the QIAmp PowerFecal Pro Kit (Qiagen) using 250 mg of fecal material from the center of each scat. A core subsample was taken from each fecal sample to ensure an even view of the bacterial community. Contamination was reduced by decontaminating the workspace and tools using 70% ethanol and bleach between each sample. Negative controls were used during each extraction batch (20 samples) to detect contaminants. DNA quantity and quality were checked using a Nanodrop (Thermo Fisher Scientific). The DNA extractions from the fecal samples had a 260/280 nm ratio of approximately ~1.8, indicating “pure DNA.” To confirm successful DNA extraction, PCR amplification of the V3‐V4 16S bacterial gene region using 341F (5′CCTAYGGGRBGCASCAG‐3′) and 806R (5′‐GGACTA CNNGGGTATCTAAT‐3′) primers was performed on a random subset of four samples from each batch. PCR reactions were carried out in a final volume of 50 μL, consisting of 10 μL MyTaq buffer, 1 μL DNA polymerase (Bioline, UK), 5 μL of each primer (final concentration 10 μM), 6 μL template DNA, and 23 μL nuclease‐free water. PCR thermal conditions included (1) initial denaturation at 95°C for 1 min, (2) 35 cycles of denaturation at 95°C for 15 s, (3) annealing at 55°C for 15 s, (4) extension at 72°C for 15 s, and (5) final extension at 72°C for 1 min. Amplification was confirmed using 1% agarose in TBE buffer stained with SYBR Safe (Life Technologies). PCR products were run along a 1‐kb ladder (Bioline) for 35 min at 90 V. Twenty microliters of each of the resulting amplicon products was loaded into 96‐well fully skirted PCR plates with a final concentration of 5–10 ng/μL and sent to Ramaciotti Centre for Genomics (University of New South Wales) for 16S V3‐V4 amplicon library preparation and Illumina MiSeq v3 2 × 300 base‐pair sequencing. The paired‐end sequence reads were demultiplexed by Ramaciotti Centre for Genomics.

### 2.2 16S Gut Microbiome Analyses

All reads were trimmed, quality filtered, merged, and denoised into ASVs using the package “dada2” version 1.26 (Callahan et al. 2016) in R version 4.2.2 (R Core Team 2022). Sequences were aligned to the Silva database (version 138; Quast et al. 2012) with dada2's “assignTaxonomy” function, which utilizes the naive Bayesian classifier method described in Wang et al. (2007). Sequences were assigned to the species level for 100% matches to the reference database using the dada2 “addSpecies” function. Sequences were then imported into the program Quantitative Insights into Microbial Ecology 2 (QIIME2‐2022.11; Caporaso et al. 2010) to create a phylogenetic tree using the fasttree algorithm (Price et al. 2009). The ASV table, taxonomy table, phylogenetic tree, and sample metadata where combined into a single object using the package “phyloseq” version 1.42 (McMurdie and Holmes 2013), designed to explore microbiome data. Singletons (sequences that occur only once in one individual), DNA from mitochondria, chloroplasts, and archaea were removed. Contaminants were identified and removed using the package “decontam” version 1.18 (prevalence method, threshold 0.5), which identifies contaminants based on their prevalence in negative controls compared to true samples (Davis et al. 2018). The magnitude of difference between the smallest and lowest library sizes was calculated to determine if rarefaction was necessary to avoid a potential loss of information (McMurdie and Holmes 2013; Weiss et al. 2017).

### 2.3 Gut Microbiome Statistics

16S data underwent a compositional transformation (sum of reads for each ASV, divided by total number of reads and made into a percentage) to show the relative abundance of dominant taxa at the different taxonomic levels to compare by location. Taxonomic levels were grouped separately (“tax_glom” function, phyloseq package). Dominant taxa were included (i.e., have an abundance greater than 1% in at least 25% of samples), with the remaining taxa placed in an “other” category using the “aggregate_rare” function in the “microbiome” package (version 1.20.0).

Alpha diversity for 16S data was explored using observed ASVs (richness) and Shannon diversity index (richness and relative abundance) with the “estimate_richness” function in phyloseq on the raw sample counts. Both metrics were tested for normality (“shapiro.test,” “stats” package version 4.3.3), homogeneity (“leveneTest”; car package, version 3.1.2), and overdispersion (“dispersiontest,” AER package version 1.2‐12). Differences in observed ASVs between location, cohort, and sex was determined using a negative binomial generalized linear model (nbGLM) to account for overdispersion in count data (“glm.nb,” MASS package version 7.3‐60.0.1). Shannon diversity index values were compared between location, cohort, and sex using a generalized linear model with gaussian link due to the positive, continuous nature of the data. A power analysis was performed using the package “InteractionPoweR” in R (version 0.2.2) with the following parameters: total sample size of *N* = 199, *α* = 0.05, assumed moderate correlations (*r* = 0.3) among predictors, and a moderate interaction effect size of *f* ^2^ = 0.05. The analysis indicated a power of only 16%, which is below the recommended 80% threshold (Brysbaert 2019). We therefore excluded interaction effects due to limited sample sizes and insufficient statistical power (Leon and Heo 2009; Zuur et al. 2009), focusing instead on the main effects of location, cohort, and sex. Holm‐Bonferroni pairwise comparisons were used to find significant differences between locations, controlling for the increased risk of Type I errors (Holm 1979) using the emmeans package (version 1.10.0).

16S beta diversity was explored using Bray–Curtis distances in phyloseq to identify potential clustering patterns in abundance and occurrence of ASVs by location, sex, and cohort (functions “distance,” “ordinate,” and “plot_ordination”). A Permutational multivariate analysis of variance (PERMANOVA; Anderson 2017) test with 9999 permutations was used to test for significant effects of location, sex, and cohort on 16S Bray–Curtis distances (“adonis2” function, vegan package, version 2.6.4). The functions “betadisper” and “permutest” were used to test assumptions of homogeneity (vegan package), in that significant PERMANOVA results were due to true differences between groups and not from individual variation within those groups.

Differential abundance testing was performed to compare 16S ASVs at the genus level between Maria Island and Woolnorth, based on similarities in phyla abundances. Each location was also compared to Fentonbury as a baseline, and Fentonbury was compared to the other common site, Kempton, due to their proximity and shared landscape characteristics. The “DAtest” package (version 2.8.0) was used to determine which differential abundance test would be most suitable based on data properties using the “pre‐DA” function to sort low‐abundance ASVs (must be present in at least two samples in minimum of 10 reads across all samples) into an “other” category, and the “testDA” function based on “location” as the predictor. DESeq2 (package version 1.38.3) with geometric means was determined as the best method to measure differential abundance. Geomeans was used to estimate the size factor by multiplying the numbers and taking the root by how many numbers there are and is the most precise when summarizing with statistics as it can account for zeros in the data. DESeq2 was run using test type “Wald,” fitType = “parametric,” sFType “poscounts” for all four locations, with *p*‐values adjusted using the Benjamini–Hochberg method (Love et al. 2014).

### 2.4 12S Diet Analyses

Methods previously outlined (McLennan et al. 2022) were used to create amplicon products of the 12S V5 region using 12Sv5F (5′‐TAGAACAGGCTCCTCTAG‐3′) and 12Sv5R (5′‐T TAGATACCCCACTATGC‐3′) primers targeting vertebrate species for all samples to capture a broad range of species, including a blocking oligonucleotide primer 12Sv5DevilB (5′‐ACCCCACTATGCTTGGCCGTAAA[C3]‐3′) to reduce Tasmanian devil host DNA amplification. PCR reactions were carried out in a final volume of 50 μL, consisting of 10 μL MyTaq buffer, 1 μL DNA polymerase (Bioline, UK), 5 μL of forward and reverse primers (final concentration 10 μM), 5 μL of blocking primer (final concentration of 100 μM), 6 μL template DNA, and 18 μL nuclease‐free water. PCR thermal conditions included (1) initial denaturation at 95°C for 10 min, (2) 35 cycles of denaturation at 95°C for 30 s, (3) annealing at 50°C for 30 s, (4) extension at 72°C for 30 s, and (5) final extension at 72°C for 10 min. Successful DNA amplification was confirmed for all samples using gel electrophoresis (1% agarose at 90 voltage for 35 min). Amplicon products were sent with Illumina overhangs to Ramaciotti Centre for Genomics for Indexing PCR and Library preparation using the Illumina MiSeq v2 sequencing platform.

Similar to 16S methods, all 12S reads were processed using the package “dada2” version 1.26 (Callahan et al. 2016) in R version 4.2.2 (R Core Team 2022). Taxonomy was assigned by matching the resulting sequence table to the NCBI reference database for mitochondrial genomes using BLASTN with an e‐value cut‐off of 1‐e20. The ASVs were further sorted based on top percent similarity. Taxonomic identification numbers (“taxid”) were obtained from NCBI's accession2taxid database before using the Entrez Direct e‐utilities (specifically “elink” and “efetch”) to retrieve the full taxonomy for each accession number. The taxonomic assignment for each ASV was examined, with percent identity cut‐offs for each taxonomic level (≥ 98% = species level, 94%–97.99% = genus level, 90%–93.99% = family level, 80%–89.99% = order level). If an ASV had more than one match based on the highest percent identity, the final taxonomic assignment was based on whether the match was confirmed to occur in Tasmania. The Atlas of Living Australia (ala.org.au) and the Department of Natural Resources and Environment Tasmania's Fauna of Tasmania webpage (nre.tas.gov.au/wildlife‐management/fauna‐of‐tasmania) were used to confirm the occurrence of diet items in Tasmania. If both classifications were found in Tasmania, the assignment went to the next highest level (e.g., from genus to family). Low‐abundance reads (≤ 10 reads in a given sample) were removed, resulting in the removal of 2760 total reads. Only potential diet items were included in the final taxonomic table, discarding primate (likely human contamination) and Dasyuridae (likely host DNA as opposed to a true diet item) from the final dataset. The ASV table, taxonomy table and sample data were combined using “phyloseq” version 1.42 (McMurdie and Holmes 2013). Contaminants were identified using negative controls during extraction and removed using the package “decontam” (prevalence method, threshold of 0.1).

### 2.5 Diet Statistics

For diet taxa analysis, we used percent frequency of occurrence (%FOO; the presence or absence of a diet item in a sample) instead of relative read abundance, which can be inflated due to sequence recovery biases of any one taxon (Deagle et al. 2019). The %FOO was calculated at each taxonomic level (class, order, family, genus, species) using custom R scripts. First, the phyloseq object was filtered based on taxonomic level, and unique ASVs at each taxonomic level were extracted and compared against the sequence table (including read counts for each sample). The sequence reads of samples greater than 0 were converted to “1,” and absent sequences received a “0.” The final data frame was grouped based on location and converted into a percentage for the final %FOO of each taxonomic group.

Similar to 16S alpha diversity, observed ASVs and Shannon diversity index were used for the 12S diet analyses and were tested for normality and homogeneity, and observed ASVs were tested for overdispersion. Observed ASVs were fitted with an nbGLM with the predictor variables of location, sex, and cohort, without interaction terms due to the limited sample sizes and insufficient statistical power (Leon and Heo 2009; Zuur et al. 2009). Pairwise comparisons to find significant differences between locations were performed using the Holm‐Bonferroni method, controlling for the increased risk of Type I errors (Holm 1979) using the emmeans package (version 1.10.0). Shannon index values were fitted with a generalized linear model with gamma link to account for a right‐skewed distribution. Similar to 16S analyses, Bray–Curtis distances were calculated for 12S beta diversity and followed by a PERMANOVA (9999 permutations) with the predictor variables of location, sex, and cohort. All three predictor variables were tested for overdispersion. To determine if there was a relationship between the gut microbiome and consumed taxa, we conducted a Mantel test (Ramette 2007) using Spearman's rank correlation. The test was performed on matrices of Bray–Curtis distances for 12S and 16S data using the ade4 package (version 1.7‐22) “mantel” function in R.

## 3 Results

### 3.1 16S Descriptive Results

Most samples (approximately 80%) yielded over 10 ng/μL of DNA. The extractions that were randomly chosen for in‐house PCR successfully amplified the 16S bacterial gene region, confirming the successful extraction of bacterial DNA from the fecal samples. A total of 79,357,372 paired‐end raw reads were obtained from 199 fecal samples, resulting in 14,318,856 successfully merged reads and a total of 3336 unique ASV after filtering and removal of contaminants. Samples were not rarefied as the library sizes only had a magnitude difference of 6.9x between the lowest (20,998) and highest (145,276) read counts.

**FIGURE 2:**
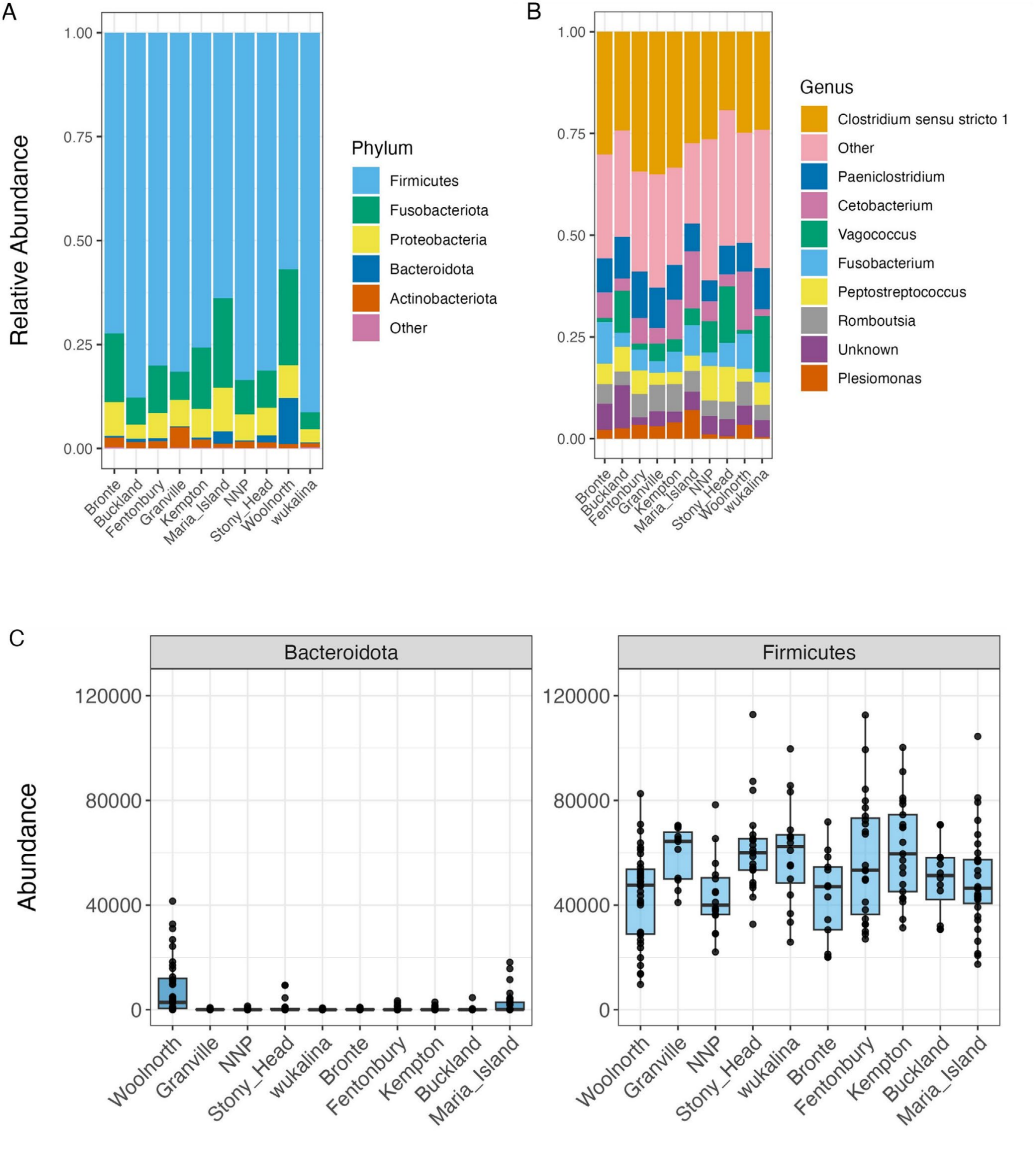
(A) The five most abundant phyla in the gut microbiome found across all 10 locations, based on relative abundance. Locations are ordered geographically from west to east. (B) The most abundant genera across all 10 locations. (C) A boxplot of abundance between the Bacteroidota and Firmicutes phyla at the 10 locations. Each point is that phylum's abundance for one sample.

The number of ASVs per sample ranged from 65 to 471. The most dominant phyla across all 10 locations were consistently Firmicutes, Fusobacteria, Proteobacteria, Actinobacteria, and Bacteroidota (Figure 2A). This included multiple taxa from the Firmicutes phylum, including one at the family level (Mycoplasmataceae), and genera *Gemella* (family Staphylococcaceae), *Paeniclostridium* (family Clostridiaceae), *Parvimonas* (genera Peptoniphilaceae), *Carnobacterium* (family Carnobacteriaceae), and *Enterococcus* (family Enterococcaceae). *Clostridium sensu stricto 1* was the most abundant genus of the Firmicutes phylum with eight ASVs (Figure 2B), five of which were identified to the species level ( *C. disporicum* , two *C. moniliforme* strains, *C. perfringens* , *C. tertium* , and *Clostridium sensu stricto 13 subterminale*). There were four other core taxa from Firmicutes that were all identified down to the species level, including *Peptostreptococcus russellii* , *Romboutsia hominis* and *R. sedimentorum*, and *Terrisporobacter mayombei*. Other core taxa were from the Fusobacteriota phylum (genus *Fusobacterium* and *Cetobacterium*) and Proteobacteria (*Escherichia‐Shigella* genus, and two ASVs of the species *Plesiomonas shigelloides* ). We examined the relative abundance of Firmicutes and Bacteroidota across all ten locations (Figure 2B). Woolnorth had the highest relative abundance of Bacteroidota (mean 0.11 ± 0.17 SD), followed by Maria Island (0.03 ± 0.06), while other locations ranged from 1.70e‐3 ± 3.60e‐3 (wukalina) to 0.02 ± 0.04 (Stony Head). There is a notably higher abundance of Bacteroidota to Firmicutes at Maria Island and Woolnorth compared to the other locations (Figure 2C).

### 3.2 16S Alpha Diversity

16S observed ASVs did not meet assumptions of normality (Shapiro–Wilks; *W* = 0.92, *p* = 3.36e‐09). We found no significant differences in variance by location (Levene's test; *F* = 0.56, *p* = 0.83), cohort (*F* = 0.59, *p* = 0.11), or sex (*F* = 0.77, *p* = 0.65). The negative binomial generalized linear model (nbGLM) found that location, cohort, and sex had a significant impact on observed ASVs. Males had fewer observed ASVs than females (*β* = −0.13 ± 0.05, *z* = −2.65, *p* = 8.00e‐3), and juveniles had more observed ASVs than adults (*β* = 0.22 ± 0.05, *z* = 4.06, *p* = 4.9e‐5). Pairwise comparisons found only two differences between locations: Woolnorth had higher observed ASVs compared to NNP (*β* = 0.39 ± 0.10, *z* = 3.78, *p* = 7.00e‐3) and Buckland (*β* = 0.38 ± 0.12, *z* = 3.30, *p* = 0.04). Shannon diversity index values met assumptions of normality (Shapiro–Wilks; *W* = 0.99, *p* = 0.57) and had no significant differences in variance by location (Levene's test; *F* = 0.77, *p* = 0.65), cohort (*F* = 1.51, *p* = 0.22), nor sex (*F* = 2.34, *p* = 0.13). Shannon index values did not significantly differ between locations. Males had lower Shannon index values than females (*β* = −0.13 ± 0.06, *z* = −2.28, *p* = 0.02), and juveniles had higher Shannon index values than adults (*β* = 0.13 ± 0.07, *z* = 2.073, *p* = 0.04). Boxplots of 16S observed ASVs and Shannon index values by location are available in Figure S1.

### 3.3 16S Beta Diversity

**FIGURE 3:**
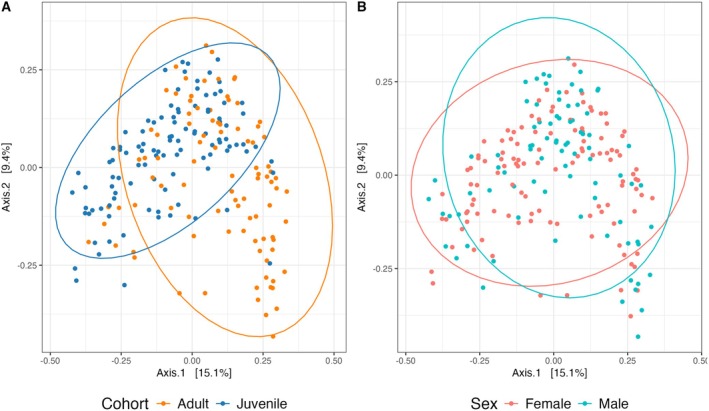
Exploration of differences in 16S Bray–Curtis distances. Includes principal coordinate analysis (PCoA) plots for cohort (A) and sex (B).

A PERMANOVA test (9999 permutations) for 16S Bray–Curtis dissimilarity showed that location explained a significant portion of variability (*R* ^2^ = 15.93%, *F* = 4.03, *p* = 1.00e‐4) and cohort (*R* ^2^ = 1.58%, *F* = 3.59, *p* = 0.0001), but no significant effect of sex (*R* ^2^ = 0.35%, *F* = 0.80, *p* = 0.75). There was borderline significance within‐sample variance for Bray–Curtis dissimilarity by location (*F* = 2.05, *p* = 0.05) and significant within‐sample variance when comparing by cohort (*F* = 7.37, *p* = 9.00e‐3), but not sex (*F* = 0.06, *p* = 0.80) suggesting that significant results may be driven by within‐group variation by cohort and individual locations. We observe different clustering patterns in the gut microbiome between juveniles and adults (Figure 3A), but there was no distinct clustering detected between males and females (Figure 3B).

### 3.4 Differential Abundance

**FIGURE 4:**
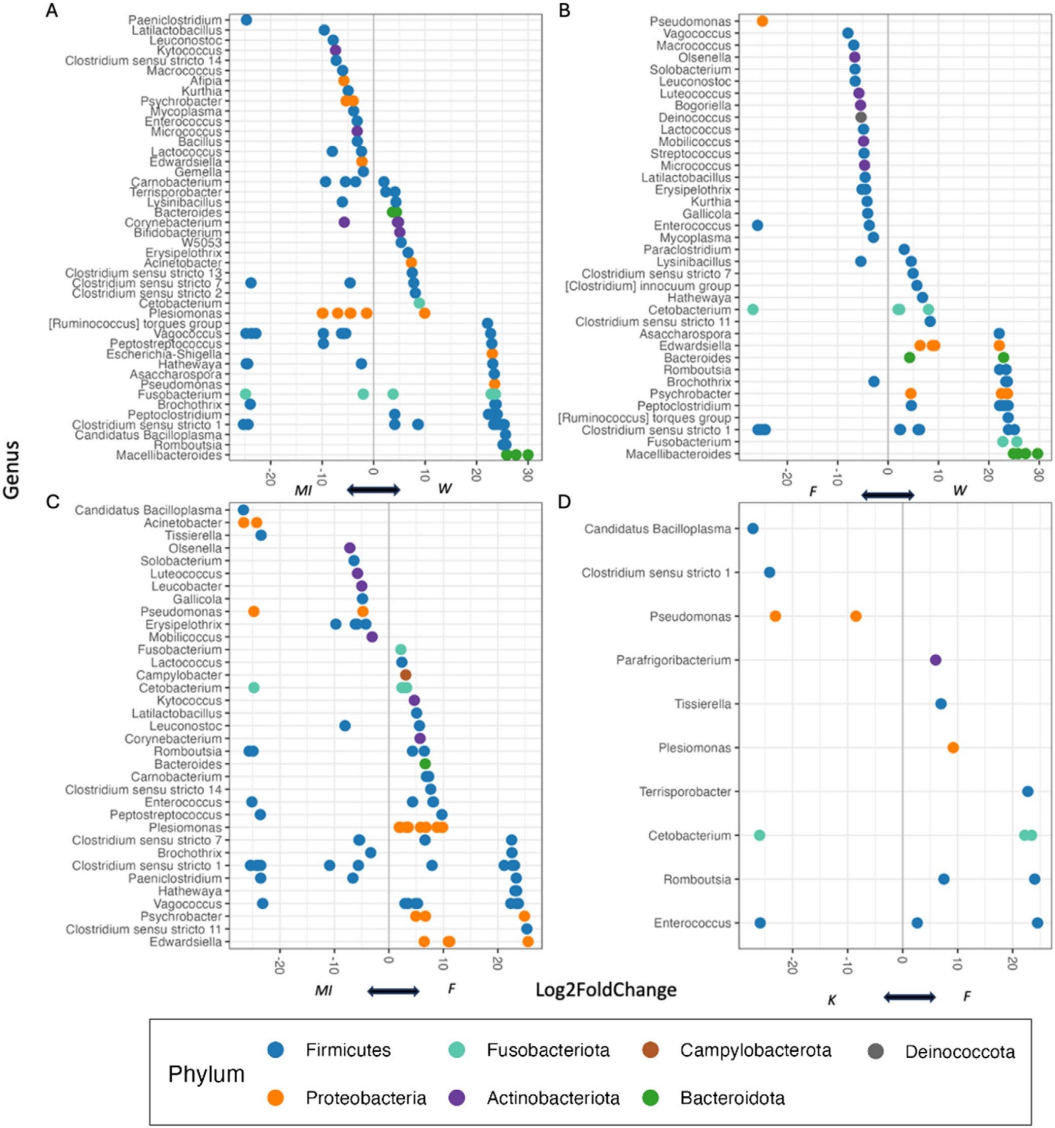
DESeq2 results showing differential abundance in ASVs as “Log2FoldChange,” which shows the relative differences between (A) Woolnorth (“W”) and Maria Island (“MI”), (B) Woolnorth and Fentonbury (“F”), (C) between Maria Island and Fentonbury, and (D) between Kempton (“K”) and Fentonbury. Each point is a unique ASV found at each of the two groups, but at different abundances. ASVs to the left of the center line are more abundant in the corresponding location found at the *x*‐axis. The closer an ASV is to a line, the less of a difference in abundance there is between the two locations. ASVs are sorted based on genus, and further differentiated to the phylum level based on color.

For differential abundance comparisons, Woolnorth, Maria Island, Fentonbury, and Kempton were chosen based on significant differences at community diversity level and their larger sample size. Woolnorth and Maria Island were statistically different based on location, in contrast to Kempton and Fentonbury that were not statistically different. DESeq2 comparisons revealed five genera that were differentially abundant to all four locations: *Clostridium sensu stricto 1*, *Pseudomonas*, *Romboutsia*, *Cetobacterium*, and *Enterococcus* (Figure 4). However, some genera had multiple differentially abundant ASVs for each location compared. Firmicutes had the highest number of differentially abundant ASVs between Maria Island (29 ASVs) and Woolnorth (23 ASVs), and eight of the genera had differentially abundant ASVs in both locations. For example, one of the most abundant genera found in devils, the *Clostridium sensu stricto 1* genus (Figure 2B) had two ASVs that were more abundant in Maria Island, and five ASVs that were more abundant in Woolnorth (Figure 4A). Maria Island did not have any differentially abundant ASVs from the Bacteroidota phylum (Figure 4A) despite having more read abundance (Figure 2A) likely due to Benjamini–Hochberg adjusted *p* values being used to account for multiple comparisons. Woolnorth had Bacteroidota ASVs that were differentially abundant between Maria Island (Figure 4A) and Fentonbury (Figure 4B), three of which belong to the *Macellibacteroides* genus and were only detected in Woolnorth for both comparisons.

### 3.5 12S Diet Results

The BLASTN match resulted in 5862 unique accession number matches across 468 unique ASVs before manual taxonomic assignment and filtering, resulting in a total of 215 total ASVs identified across all samples (*n* = 198). These ASVs belonged to class Mammalia (mammals, *n* = 126 ASVs), Aves (birds, *n* = 59 ASVs), Actinopteri (ray‐finned fishes, *n* = 19 ASVs), Reptilia (8 ASVs) and Amphibia (3 ASVs). Of all 215 ASVs, 30 could confidently be assigned to species level (13.95%), 96 to genus level (44.65%), 73 to family level (33.95%), and 16 to order level (7.44%).

The Macropodidae family of marsupials was detected in all 198 samples found across all 10 locations (Table 1). Other common mammalian orders included Order Artiodactyla (hooved mammals), which were detected across all 10 locations with 100% FOO in Bronte, Granville, and wukalina. At least one livestock group was detected in all these locations: sheep (*Ovis* sp.), cattle (*Bos* sp.), goats (*Capra* sp.), and pigs (Suidae family). The detection of livestock in scat samples may not represent prey taxa but instead be from campsites (i.e., pork sausages) at these locations or devils consuming scat from these individuals or feeding on deceased carcasses. Other mammalian orders that were detected include Rodentia (rats and mice), Lagomorpha (rabbits), Carnivora (eared seals, cats, and dogs), and Chiroptera (bats).

**TABLE 1:** The %FOO at the class level, which is the total presence/absence samples with at least one diet item, divided by the total number of samples at that location and expressed as a percentage.

Location	Mammalia	Aves	Reptilia	Actinopteri	Amphibia	*N*	Month
Bronte	100	23.08	—	—	—	13	May/June
Buckland	100	16.67	33.33	—	—	12	March
Fentonbury	100	37.50	—	4.17	—	24	May
Granville	100	54.55	27.27	—	—	11	June
Kempton	100	35.00	35.00	—	—	20	May
Maria Island	100	26.67	—	40.00	3.33	30	June
NNP	100	50.00	—	12.50	—	16	April
Stony Head	100	28.57	19.05	14.29	14.29	21	March
Woolnorth	100	25.71	20.00	—	—	35	July
Wukalina	100	37.50	18.75	6.25	—	16	February

*Note:* For example, taxa belonging to the class “Mammalia” were found in 100% of samples across all 10 locations, while taxa belonging to the class “Amphibia” were only seen at two locations in a smaller percentage of samples. Sample size (*N*) and time of collection (Month) are provided.

Of Class Aves (birds) consumption was detected in all 10 locations. Order Passeriformes was the most common, followed by Psittaciformes and Galliformes. Class Actinopteri (ray‐finned fishes) was mostly detected on Maria Island (40%FOO, Table 1), which also had the most Actinopteri orders consumed. Class Reptilia was detected in six locations and consisted of orders Squamata (scaled reptiles) and Testudines (turtles). Only one Amphibia order was detected (Anura) at two locations (Table 1). A complete list of %FOO by taxonomic group is available in Table S1.

### 3.6 12S Alpha Diversity

Alpha diversity was investigated using observed ASVs and Shannon's diversity index for 12S diet taxa. Observed ASVs did not meet assumptions of normality (Shapiro–Wilk; *W* = 0.91, *p* = 0.02), and there was significant evidence that variances were not evenly dispersed among locations (Levene's test; df = 9, *F* = 3.33, *p* = 8.37e‐4) so a negative binomial generalized linear model (nbGLM) was used. The model of best fit based on AIC values included both location and cohort as explanatory variables, so sex was excluded from the final model. There was no significant effect of cohort on the observed ASVs (nbGLM; *β* = 0.13, SE = 0.07, *z* = 1.86, *p* = 0.06). Pairwise comparisons by location showed significant differences in observed ASVs between Kempton and three locations: NNP (*p* = 0.03), Stony Head (*p* = 1.20e‐3), and Maria Island (*p* = 7.50e‐3), and between Stony Head and Woolnorth (*p* = 4.94e‐2).

Shannon's diversity index values did not meet assumptions of normality (Shapiro–Wilk; *W* = 0.99, *p* = 0.04) but did show evidence of even dispersion among locations (Levene's test; df = 9, *F* = 1.4, *p* = 0.23), justifying the use of the non‐parametric generalized linear model with gamma link to account for the slight right skew in the data. The model of best fit based on AIC values only included location as an explanatory variable, so Cohort and Sex were excluded from the final model. Boxplots of 12S observed ASVs and Shannon index values by location are available in Figure S2.

### 3.7 12S Beta Diversity

**FIGURE 5:**
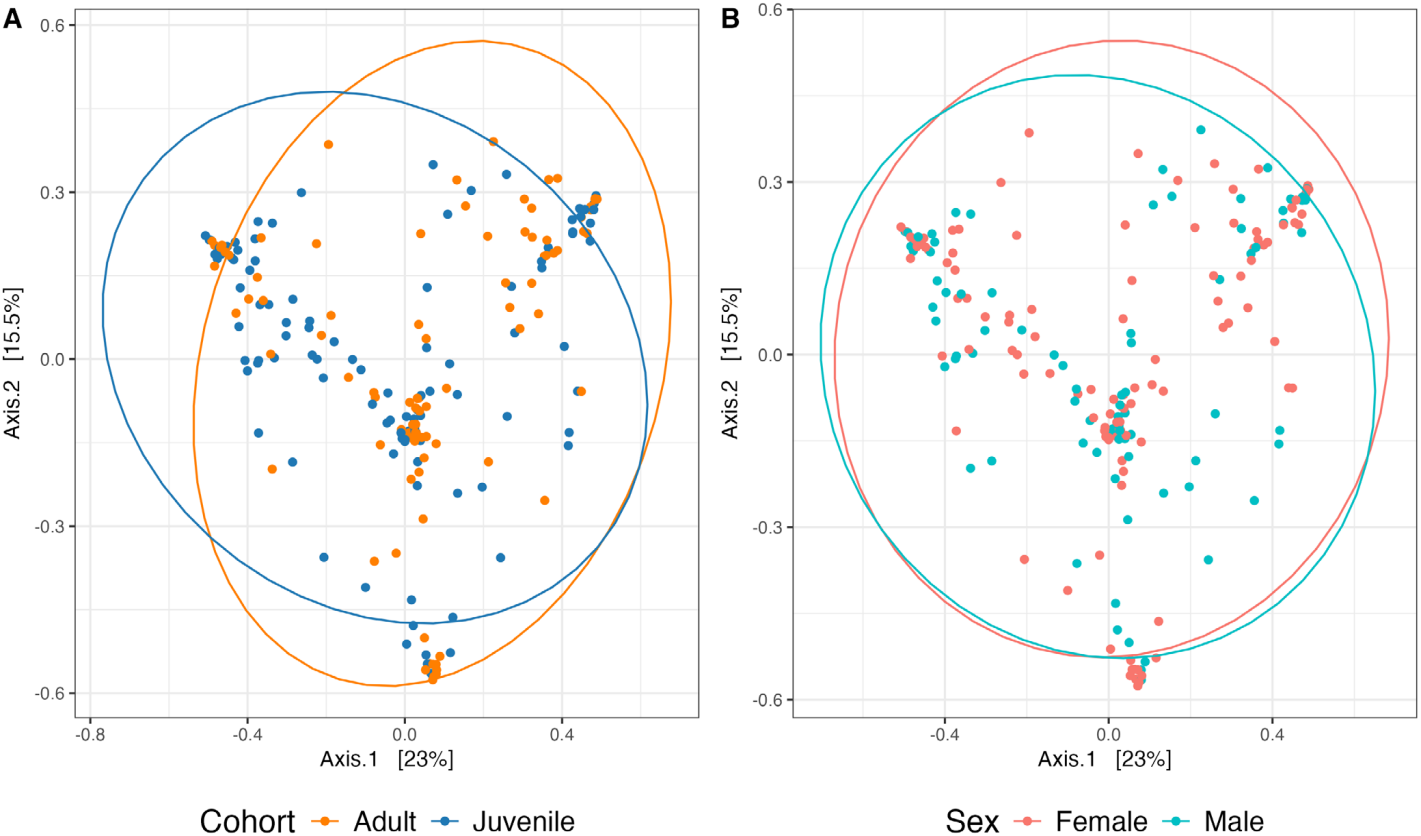
Exploration of differences in 12S Bray–Curtis distances. Includes principal coordinate analysis (PCoA) plots for cohort (A) and sex (B).

PERMANOVA tests (9999 permutations) for diet Bray–Curtis dissimilarity showed that location explained a significant portion of variability (*R* ^2^ = 17.28%, *F* = 4.36, *p* = 1.00e‐4). There was no effect of cohort (*R* ^2^ = 0.39%, *F* = 0.88, *p* = 0.52) or sex (*R* ^2^ = 0.36%, *F* = 0.81, *p* = 0.60). There was no significant within‐sample variance of diet patterns by location (*F* = 1.74, *p* = 0.08), suggesting that significant PERMANOVA results are due to true variations between locations. In contrast to 16S beta diversity (Figure 3), we do not see distinct beta diversity clustering patterns between juveniles and adults in 12S data (Figure 5A). Clustering differences are also not evident between males and females in diet taxa consumed (Figure 5B).

A Mantel test comparing the Bray–Curtis matrices showed that there was a statistically significant but weak positive correlation between the 16S and 12S datasets (*r* = 0.1095, *p* = 0.002), suggesting some relationship between the 16S and 12S datasets.

## 4 Discussion

We aimed to determine if there were differences in Tasmanian devil gut microbiome richness, diversity, and community structure across Tasmania. Furthermore, we sought to understand if location, age cohort, and sex influence the gut microbiome, and if there is a strong relationship between the 12S diet and any gut microbiome differences. While the location was found to be a significant source of variability in both 16S and 12S communities, vertebrate diet only explained a small amount of gut microbiome variability. Juvenile devils were shown to have diverging gut microbiome communities from adults along with higher bacterial diversity, but there was no strong evidence that juveniles were consuming different vertebrates. We also showed that each location had a higher amount of Firmicutes to Bacteroidota phyla in the gut microbiome similar to what has been previously described for this species (Cheng et al. 2015; Chong et al. 2019). Notably, there was a higher relative and absolute abundance of Bacteroidota at Maria Island and Woolnorth compared to the other locations.

Microbes can adapt and diversify in response to different environments (Wani et al. 2022; Petersen et al. 2023) while maintaining the same functions beneficial to the host (Tian et al. 2020). The detection of multiple ASVs for the same genus at different abundances between locations may indicate functional redundancy. Functional redundancy is proposed to be a sign of a stable and resilient gut microbiome suggesting that certain taxa hold the same biological role for the host, conserving important gut functions including metabolic homeostasis (Thursby and Juge 2017) and immune response (Islam et al. 2022). This redundancy further supports the “insurance policy hypothesis” that the microbiome can still perform important host functions among different environments (Rosenberg 2021).

In our dataset, there were three ASVS from the genus *Macellibacteroides* Bacteroidota phylum, Tannerellaceae family) that were differentially abundant in Woolnorth compared to Maria Island and Fentonbury. *Macellibacteroides* is a recently characterized bacterial genus with one known species (*Macellibacteroides fermentans*) isolated during wastewater treatment (Jabari et al. 2012) and can withstand high alkaline environments (Rout et al. 2017). Hippopotamus ( *Hippopotamus amphibius* ) were found to be dominated by *Macellibacteroides* sp. in a wild population (Dutton et al. 2021). Little is known about this bacterial species, but the family Tannerellaceae is associated with propionate production, which is a short‐chain fatty acid (SCFA) associated with dietary fiber digestion (Van den Abbeele et al. 2022). While it is possible devils at Woolnorth may have a higher capacity for this process, it may be a short‐term effect from consuming the digestive tract of organisms equipped with bacteria for dietary fiber digestion, such as livestock and native herbivores. Functional profiling and metabolomics to detect SCFA production are necessary to further understand the differential abundance of this genus in Woolnorth devils.

Our diet analysis results among the 10 locations across Tasmania confirm that devils consume a wide variety of vertebrates and support their role as opportunistic carnivores and facultative scavengers (Andersen et al. 2017). As expected, all locations had high amounts of native marsupial taxa. We also documented a wide variety of bird species consumed, with most marine taxa occurring at coastal locations (Maria Island, Stony Head, NNP, Woolnorth). Sampling locations that were near or on farmland (all locations except Maria Island and NNP) had high amounts of livestock consumed, including goats, sheep, and cattle. In contrast, Maria Island devils mainly consumed native marsupial taxa, shorebirds, and fish, which is similar to previous findings into the diet of Maria Island devils (McLennan et al. 2022). Family Otariidae (eared seals) was detected in nine Maria Island samples (30.00%FOO), which is likely to be from scavenging a deceased individual. Two livestock genera were found in Maria Island samples: Sheep (*Ovis* spp.) in four out of 30 devils on Maria Island (13.33%FOO) and cattle (*Bos* spp.) in two devils (6.67%FOO). There are no livestock managed on Maria Island, so these findings are likely food sources from the different campgrounds on the island. We therefore recommend that the Tasmania Parks and Wildlife Service continue to emphasize to visitors the importance of minimizing the availability of anthropogenic food to protect the natural diets of Tasmanian devils.

Tasmania has had multiple introductions of non‐native species that are considered pests and damage natural ecosystems. We found invasive taxa including European rabbits ( *Oryctolagus cuniculus* ) and genus *Capra* spp. and *Sus* spp., which may be feral goats and pigs, respectively. We also found evidence of domestic cat consumption (*Felis* spp.) in three locations at low occurrences. Feral cats are a concern due to their impact on endemic wildlife. Higher cat abundances have been found in areas with devil population declines (Cunningham et al. 2020). Restored Tasmanian devil populations are expected to decrease the feral cat population (Hunter et al. 2015). However, it is unclear whether cat DNA detected in our samples was from direct predation and scavenging, or from consuming cat fecal matter. While invasive species may acquire a functional ecological role as a main prey source for native carnivores (Buenavista and Palomares 2018), the low %FOO of these taxa in our study does not provide strong evidence that they are becoming a main food source for Tasmanian devils.

Age cohort was found to have a significant effect on the gut microbiome, despite no strong evidence that juveniles have a different diet composition compared to adults. In humans, age‐related gut microbiome changes are highly variable and influenced by many environmental and individual factors, but a decrease in diversity and an increase in richness is usually documented (Ghosh et al. 2022). This trend was observed in our study, where juveniles were found to have higher gut microbial diversity compared to adult devils. Similar trends in microbial differences with aging have been observed in other species, including increased diversity in juvenile pigs (Lim et al. 2019), decreased diversity in geriatric marmosets ( *Callithrix jacchus* ) (Reveles et al. 2019), and large changes in dominant phyla in rhesus macaques ( *Macaca mulatta* ) from juvenile to geriatric life stages (Adriansjach et al. 2020). Immune system repertoires decrease in diversity with age (Simon et al. 2015), as documented in species including Tasmanian devils (Cheng et al. 2017) and European badgers ( *Meles meles* ; Beirne et al. 2016). The link between immune function and gut microbial diversity (Hooper et al. 2012; Shao et al. 2023) may be a possible explanation for gut microbiome diversity differences seen in our study.

16S community structure was the same between males and female devils, but females had overall higher bacterial richness and diversity. In humans, females also have higher bacterial richness compared to men post‐puberty (Sisk‐Hackworth et al. 2023) perhaps due to higher estrogen levels (Valeri and Endres 2021; Korpela et al. 2021). Sexual dimorphism in gut microbiomes may be caused by the dimorphism witnessed in the adult immune system (Klein and Flanagan 2016). These gut microbiome changes post‐puberty may also explain the microbial community structure differences seen between adult and juvenile devils in this study.

DFTD mainly impacts adult devils at breeding age (Woods et al. 2018) and as a result, there are more devils less than 1 year of age at sampling locations. Maria Island and Woolnorth are DFTD‐free locations and had a higher number of adults in this study (19 and 30, respectively) while the other eight locations in this study are impacted by DFTD and have more juveniles. This is a challenge when trying to understand the compounding factors of age cohort and location. Fentonbury and Kempton are two sites with similar sample sizes and balanced cohort sizes; they are also located in the same geographic area and were found to have no differences in alpha or beta gut microbiome pairwise comparisons and are known to have high gene flow between them (Schraven et al. 2024). There is evidence in our study that juveniles have a different 16S community composition than adults, and this may be driving differences in microbial communities between locations. Although it is hard to tease apart the impact of location and age cohort on gut microbiome beta diversity differences, differences in age cohort are important to consider.

Environmental factors other than diet can influence gut microbiome communities. Different locations can vary in the type of microbial communities present due to different climates (Finkel et al. 2011), precipitation (Zhou et al. 2018), and differences in soil–plant interactions (Classen et al. 2015). Microorganisms in soil may be able to influence the gut microbiome of mammals (Wall et al. 2015). Only a small amount of soil microbes was found to be adopted by the Tibetan macaque ( *Macaca thibetana* ) gut microbiome (Xu et al. 2022). However, exposure to soil microbes was found to assist in the rewilding of captive ring‐tailed lemur ( *Lemur catta* ) gut microbiomes (Bornbusch et al. 2022). Agricultural practices may affect soils by altering microbial community and function. Cultivated soils were found to have decreased richness due to declines in rare microbes in New South Wales, Australia (Pino et al. 2023). Organic farming has also been found to alter soil microbial diversity and activity in Finland (Peltoniemi et al. 2021). While there is currently no available data comparing soil microbiomes by location for Tasmania, there are increased efforts to characterize soil microbial communities across Australia (Birnbaum et al. 2024). Tasmanian devils on or near land that is heavily cultivated may result in differences in gut microbial diversity due to alterations in soil microbial communities and may explain some of the variation captured in our study.

Parasites are known to alter the gut microbiome in humans by impacting the microbial composition and important functions including nutrient metabolism by the host (Beyhan and Yıldız 2023). Parasites of the Tasmanian devil remain largely understudied (Wait, Peck, et al. 2017). Novel genotypes of *Cryptosporidium* and *Giardia* have been described, and notable increases in parasite prevalence in wild devils compared to captive individuals have been found (Wait, Fox, et al. 2017). In humans, both *Giardia* and *Cryptosporidium* infections are associated with gut microbial dysbiosis (Fekete et al. 2021; Naveed and Abdullah 2021). Mice infected with *Giardia* had a shift in the abundance of main phyla, with an increase in Firmicutes and a decrease in Bacteroidota (Bartelt et al. 2017). In addition to *Cryptosporidium* and *Giardia*, other parasites, including *Baylisascaris tasmaniensis*, *Taeniid* spp., coccidia, and *Strongylid* spp., were found to vary in prevalence between different wild, free‐range captive, and captive Tasmanian devil populations (Wait 2022). Based on evidence that parasites impact the gut microbiome, differences in gut microbial richness and diversity between locations may be driven by differences in parasite species and abundance.

Higher levels of Bacteroidota in Maria Island and Woolnorth devils were not explained by diet. However, Bacteroidota are known to metabolize polysaccharides, with example sources including plants, algae, and chitin that can be obtained from crustaceans and insects (Flint et al. 2008; Elieh‐Ali‐Komi and Hamblin 2016). These taxa were not targeted by our 12S vertebrate primer and so were not detected in this study, but the similarities between Maria Island and Woolnorth may be driven by consumed taxa not captured in our study. Our results provide a strong foundation for understanding the vertebrate component of the devil diet but may not capture the full dietary range, which should be explored in future studies. Interestingly, Maria Island and Woolnorth are also the only disease‐free sites with abundant devil populations included in this study (Forestier Peninsula is another disease‐free site in Tasmania), where high competition for food may play a role in the elevated Bacteroidota levels. In particular, the offal pits at Woolnorth, where devils consume highly degraded cow carcasses, might contribute to this higher abundance. The spotted hyena ( *Crocuta crocuta* ), another known scavenger, has dominant levels of Bacteroidota similar to those seen in our study (Chen et al. 2020) highlighting the potential impact that scavenging and subsequent exposure to decomposition‐related bacteria may have on the gut microbiome.

A potential limitation with our comparisons of diet species is that variations in weather and seasonality may impact the %FOO detected. It was previously shown that devils on Maria Island consume different prey items between the summer and winter months (McLennan et al. 2022). Due to the availability of field staff, sample collections across Tasmania occurred over 5 months (February to July), which may lead to uneven comparisons of diet items detected across different locations. Species that hibernate, such as short‐beaked echidnas ( *Tachyglossus aculeatus setosus* ; Nicol and Andersen 2002), may be underrepresented during the winter. Migratory bird species, such as short‐tailed shearwaters (*Ardenna tenuirostris*), which occur in Tasmania during the spring and early autumn, may only be available as prey at certain times of the year (Bool et al. 2024). Despite these potential seasonal effects, the observed %FOO still matched our expectations in that mammals and birds dominated as diet items (Pemberton et al. 2008; Andersen et al. 2020).

Another caveat is that with the metabarcoding method, we were unable to differentiate between direct predation, scavenging, and secondary consumption (Deagle et al. 2019). For instance, samples with feral cat consumption may also include birds, small marsupial species, and other cat prey items that were then consumed by the devil eating the cat scat or cat digestive tract. To mitigate the potential influence of primer specificity and variable amplification with our metabarcoding method, we used primers with broad taxonomic coverage and applied filtering criteria to reduce false positives (Zinger et al. 2019). We also used %FOO to avoid issues with differential amplification success across taxa (Deagle et al. 2019). However, our read assignments are limited to the sequences available within the NCBI 12S database, which may result in the exclusion of taxa not represented in the reference database (Zinger et al. 2019). This limitation could reduce the resolution of taxonomic assignments, potentially restricting some reads to higher taxonomic levels rather than species‐level identification. Expanding reference databases with more comprehensive sequencing of Australian species would improve taxonomic resolution in future studies.

Our study suggests that diet may not be the sole or dominant factor driving gut microbiome variability in Tasmanian devils, but incorporating non‐vertebrate dietary components in future studies may offer a more complete understanding. We additionally provide a baseline of individual gut microbiomes to allow for longitudinal studies to occur in Tasmanian devils. Of specific interest is that Maria Island and Woolnorth are sites that do not have DFTD and were found to have different microbiome compositions when compared with beta diversity and differential abundance analysis. Further research is needed to determine if there is a relationship between DFTD presence and the host gut microbiome in Tasmanian devils. To date, this is the first range‐wide gut microbiome study of an endangered animal threatened with disease, helping to understand the relationship between gut microbiome and diet to inform conservation management decisions.

## Author Contributions

**Meadhbh M. Molloy:** data curation (lead), formal analysis (lead), investigation (lead), methodology (lead), writing – original draft (lead), writing – review and editing (equal). **Elspeth A. McLennan:** formal analysis (supporting), investigation (equal), methodology (supporting), project administration (supporting), supervision (equal). **Samantha Fox:** resources (lead), writing – review and editing (supporting). **Katherine Belov:** conceptualization (equal), funding acquisition (equal), supervision (equal). **Carolyn J. Hogg:** conceptualization (equal), investigation (equal), project administration (lead), supervision (equal), writing – review and editing (equal).

## Conflicts of Interest

The authors declare no conflicts of interest.

## Supporting information


Data S1

## Data Availability

Code, raw sequence data and metadata for the 16S and 12S reads (fastq.gz files) on Dryad: http://datadryad.org/stash/share/9TPRW3nObM9AVpFQfopCILrpMp3FtNoDcra5BSP_ODs (for journal review). https://doi.org/10.5061/dryad.qz612jmrj.
