# Supplementary material for: Range‐Wide Assessment of the Tasmanian Devil Gut Microbiome
Source: Ecol Evol. 2025 May 4;15(5):e71196. doi: 10.1002/ece3.71196 (PMC12050263; doi:10.1002/ece3.71196)
Supplement: Supplementary file 1 — Data S1 [file ECE3-15-e71196-s001.docx]

**Range-wide assessment of the Tasmanian devil gut microbiome**

Meadhbh M. Molloy^1,2^, Elspeth A. McLennan^1^, Samantha Fox^3,4^, Katherine Belov^1,5^, Carolyn J. Hogg^1,5*^

**Supplementary Material**


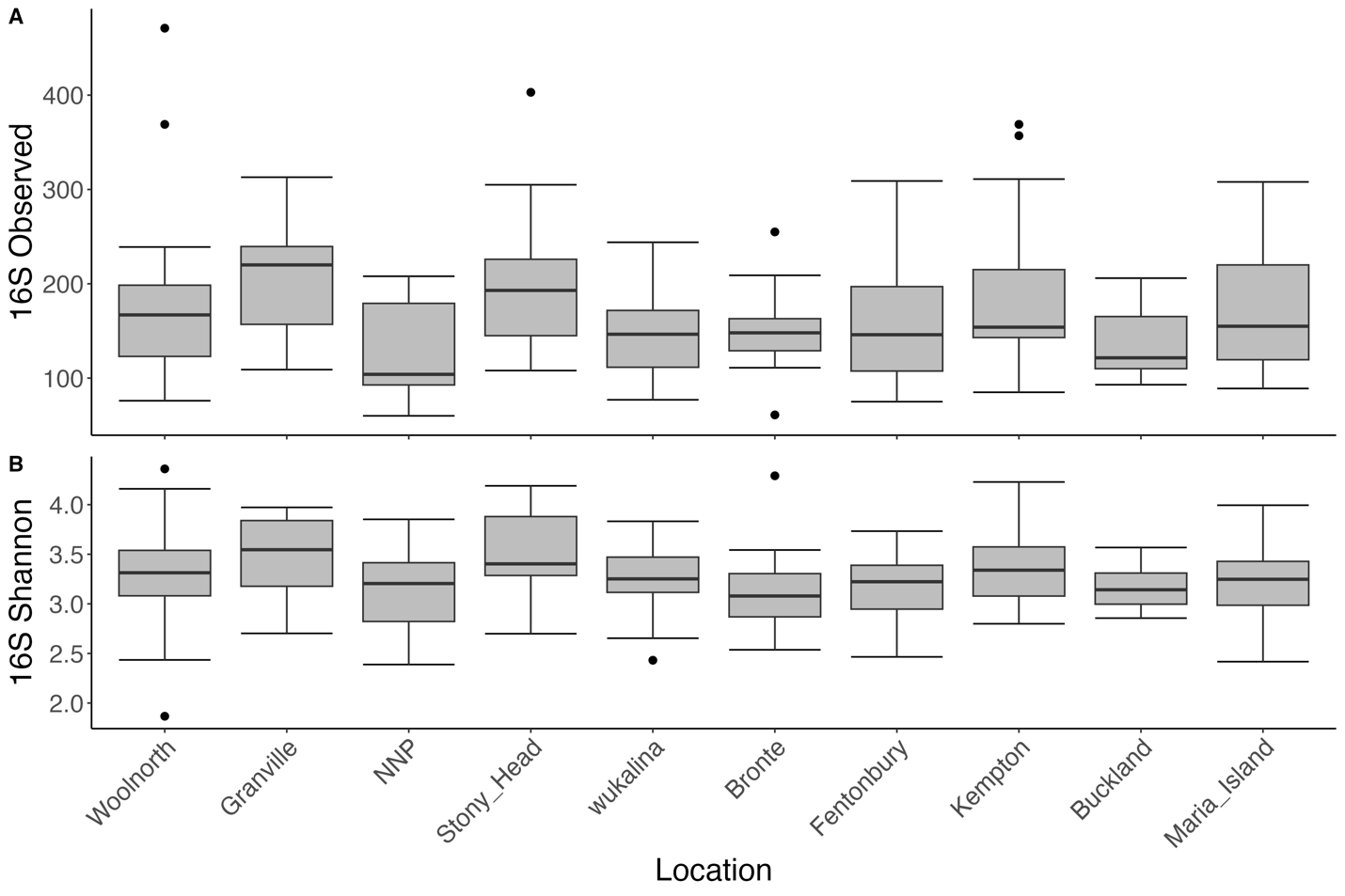


**Figure S1**. Gut microbiome alpha diversity across locations. Observed ASVs (A) are compared for gut microbiome ASV richness. Shannon index accounts for both presence/absence and abundance of ASVs (B). Higher Shannon index values indicate higher diversity and/or more even distribution of abundance among the different ASVs.


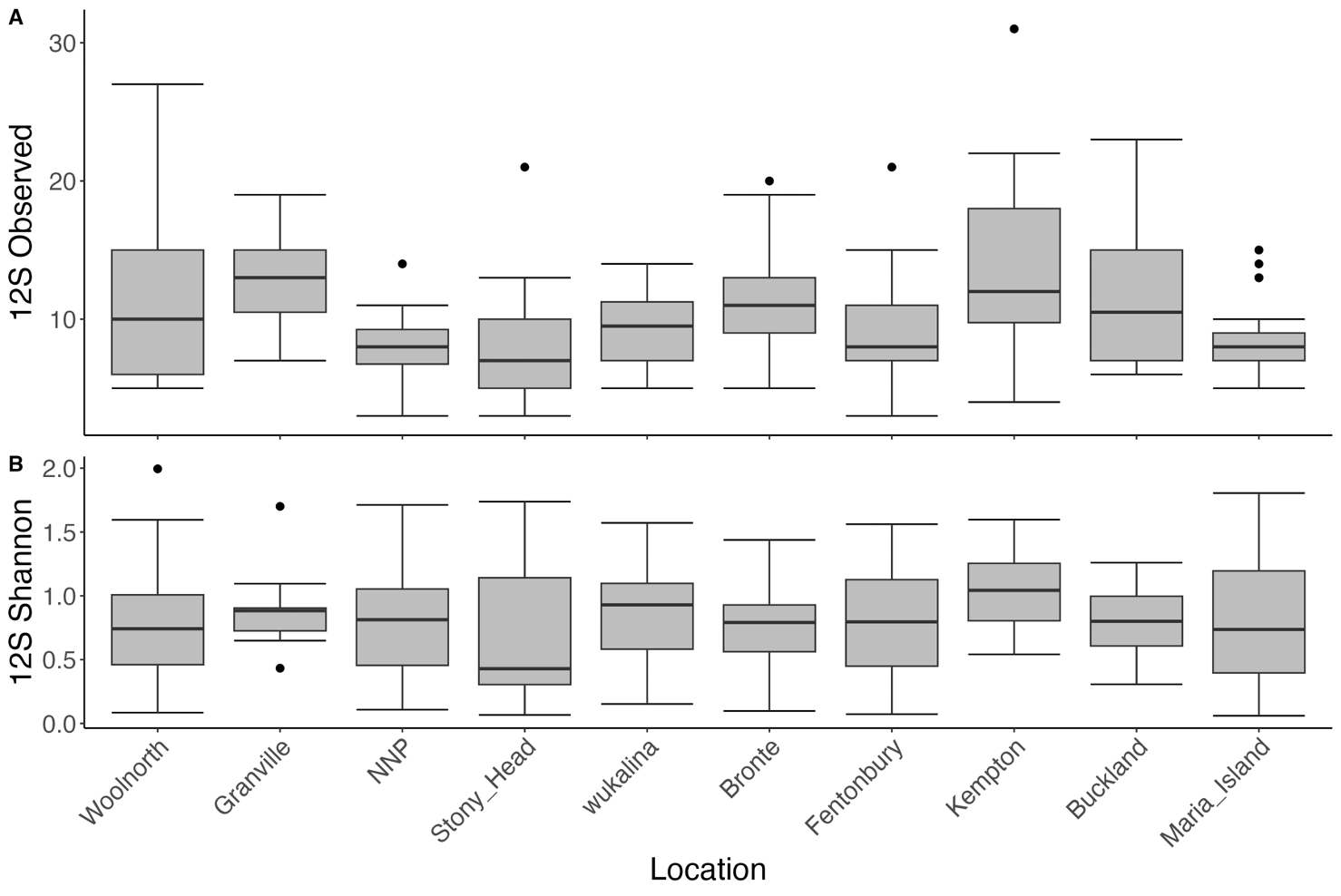


**Figure S2.** Diet alpha diversity across locations comparing observed ASVs (A) which refers to the number of unique diet taxa found, and Shannon index (B) which accounts for both presence/absence and abundance of diet taxa. Higher Shannon index values indicate higher diversity and/or more even distribution of abundance among the different diet taxa.

**Table S1.** Percent Frequency of Occurrence (%FOO) of diet items for all ten locations. Diet items are sorted by class and separated by order. The highest level of taxonomic precision available is provided.

|  | **Bronte** | **Buckland** | **Fentonbury** | **Granville** | **Kempton** | **Maria Island** | **NNP** | **Stony Head** | **Woolnorth** | **wukalina** |
| --- | --- | --- | --- | --- | --- | --- | --- | --- | --- | --- |
| ***Mammalia*** | **100** | **100** | **100** | **100** | **100** | **100** | **100** | **100** | **100** | **100** |
| Order Artiodactyla | 100 | 92 | 79 | 100 | 95 | 17 | 25 | 67 | 97 | 100 |
| Bovidae family | 100 | 92 | 79 | 100 | 95 | 13 | 19 | 62 | 97 | 100 |
| *Bison spp.* | — | — | — | — | — | — | — | — | 11 | — |
| *Bos spp.* | 15 | 8 | 8 | 36 | 15 | 7 | — | 29 | 63 | 13 |
| *Capra spp.* | — | 8 | — | — | 10 | — | — | — | 9 | — |
| *Ovis spp.* | 100 | 92 | 79 | 100 | 95 | 13 | 13 | 57 | 97 | 100 |
| Cervidae family | 8 | 17 | 17 | 18 | 30 | — | 6 | 10 | 17 | — |
| *Dama dama* | 8 | — | 17 | — | 10 | — | 6 | 10 | 3 | — |
| Suidae family | — | 8 | — | — | — | 7 | — | — | — | — |
| *Sus spp.* | — | 8 | — | — | — | 7 | — | — | — | — |
| Order Carnivora | 8 | — | 4 | — | — | 30 | — | 10 | — | — |
| *Canis spp.* | — | — | 4 | — | — | — | — | — | — | — |
| *Felis spp.* | 8 | — | 4 | — | — | — | — | 10 | — | — |
| Otariidae family | — | — | — | — | — | 30 | — | — | — | — |
| *Arctocephalus spp.* | — | — | — | — | — | 10 | — | — | — | — |
| Order Chiroptera | — | 17 | 4 | 55 | — | — | — | — | 6 | — |
| Order Diprotodontia | 100 | 100 | 100 | 100 | 100 | 100 | 100 | 100 | 100 | 100 |
| Macropodidae family | 100 | 100 | 100 | 100 | 100 | 100 | 100 | 100 | 100 | 100 |
| *Macropus spp.* | 92 | 100 | 100 | 100 | 100 | 100 | 100 | 100 | 100 | 94 |
| *Petaurus spp.* | — | 8 | 4 | 9 | — | 3 | 6 | — | — | — |
| Phalangeridae family | 23 | 17 | 17 | — | 30 | 13 | 13 | 24 | 6 | 38 |
| *Trichosurus spp.* | 23 | 17 | 17 | — | 30 | 10 | 13 | 24 | 6 | 38 |
| *Potorous tridactylus* | — | — | — | — | — | 7 | — | 5 | — | — |
| *Pseudocheirus ssp.* | 54 | — | 4 | 36 | 5 | 3 | 19 | 24 | 6 | — |
| *Vombatus ursinus* | 54 | 33 | 17 | 9 | 40 | 23 | — | 14 | — | 38 |
| Order Lagomorpha | — | 17 | 4 | 18 | 15 | — | — | 19 | — | — |
| *Oryctolagus cuniculus* | — | 17 | 4 | 18 | 10 | — | — | 19 | — | — |
| *Lepus spp.* | — | — | — | — | 5 | — | — | — | — | — |
| Order Monotremata | — | — | — | — | — | — | — | 10 | — | 13 |
| *Tachyglossus spp.* | — | — | — | — | — | — | — | 10 | — | 13 |
| Order Peramelemorphia | — | — | — | — | — | — | 13 | — | — | — |
| *Isoodon spp.* | — | — | — | — | — | — | 13 | — | — | — |
| Order Rodentia | 85 | 100 | 92 | 100 | 100 | 100 | 88 | 95 | 86 | 94 |
| Muridae family | — | 8 | 4 | — | 10 | 10 | 13 | 24 | 3 | — |
| *Mus musculus* | — | 8 | — | — | 10 | — | 6 | 14 | — | — |
| *Rattus spp.* | — | — | 4 | — | — | 10 | 6 | 5 | 3 | — |
| ***Aves*** | **23** | **17** | **38** | **55** | **35** | **27** | **50** | **29** | **26** | **38** |
| Order Accipitriformes | — | — | — | — | — | — | 13 | — | — | — |
| *Accipiter ssp.* | — | — | — | — | — | — | 13 | — | — | — |
| Order Anseriformes | — | — | — | — | — | — | 13 | — | — | 13 |
| Anatidae family | — | — | — | — | — | — | 13 | — | — | 13 |
| Order Charadriiformes | — | 8 | 4 | — | 5 | 13 | 6 | — | 6 | 6 |
| *Charadrius mongolus* | — | — | — | — | — | — | — | — | 3 | 6 |
| Laridae family | — | 8 | — | — | — | 7 | — | — | 3 | 6 |
| *Chroicocephalus spp.* | — | 8 | — | — | — | 3 | — | — | 3 | 6 |
| *Larus dominicanus* | — | — | — | — | — | 3 | — | — | — | — |
| Turnicidae family | — | — | 4 | — | 5 | 7 | 6 | — | — | — |
| *Turnix spp.* | — | — | 4 | — | 5 | 7 | 6 | — | — | — |
| Order Columbiformes | — | 8 | — | — | — | 3 | 6 | — | — | — |
| *Geopelia spp.* | — | 8 | — | — | — | 3 | 6 | — | — | — |
| *Falco peregrinus* | — | — | — | — | 5 | — | — | — | — | — |
| Order Galliformes | — | — | 8 | 9 | 5 | 3 | — | 10 | 3 | — |
| Cracidae family | — | — | — | — | — | — | — | 5 | — | — |
| Phasianidae family | — | — | 4 | 9 | 5 | — | — | — | — | — |
| *Gallus gallus* | — | — | 4 | — | — | — | — | — | — | — |
| Order Gruiformes | — | — | — | — | — | — | 6 | — | — | 6 |
| *Fulica spp.* | — | — | — | — | — | — | — | — | — | 6 |
| *Lewinia spp.* | — | — | — | — | — | — | 6 | — | — | — |
| Order Passeriformes | 15 | 17 | 29 | 45 | 10 | 17 | 19 | 14 | 11 | 19 |
| Acanthizidae family | — | — | — | 9 | — | — | — | — | — | — |
| *Piranga spp.* | — | — | 4 | — | — | — | — | — | — | — |
| *Corvus spp.* | — | — | 13 | — | — | — | 6 | 5 | 3 | — |
| *Hirundo spp.* | — | — | — | — | — | — | 6 | — | — | — |
| Meliphagidae family | — | 8 | 4 | — | 10 | 13 | 6 | — | — | — |
| *Acanthorhynchus tenuirostris* | — | — | — | — | — | 7 | — | — | — | — |
| *Pachycephala spp.* | — | — | — | 9 | — | 0 | — | — | 3 | — |
| *Petroica spp.* | — | — | — | — | — | 0 | — | — | 3 | — |
| *Sturnus vulgaris* | — | — | 4 | — | — | 3 | — | 5 | — | 6 |
| *Turdus spp.* | — | — | — | 18 | — | — | — | 5 | — | — |
| *Zosterops spp.* | — | — | 4 | — | — | — | — | — | — | 6 |
| Order Procellariiformes | — | — | — | — | — | 3 | — | — | — | — |
| *Ardenna spp.* | — | — | — | — | — | 3 | — | — | — | — |
| Order Psittaciformes | 8 | — | — | — | 15 | 10 | 13 | 5 | 6 | — |
| *Nymphicus spp.* | — | — | — | — | — | — | 6 | — | — | — |
| Psittacidae family | 8 | — | — | — | 15 | 10 | 6 | 5 | 6 | — |
| Order Sphenisciformes | — | — | — | — | — | — | — | 5 | — | — |
| *Eudyptes spp.* | — | — | — | — | — | — | — | 5 | — | — |
| Order Suliformes | — | — | 4 | — | — | — | — | — | 3 | — |
| *Phalacrocorax spp.* | — | — | 4 | — | — | — | — | — | 3 | — |
| ***Actinopteri*** | **—** | **—** | **4** | **—** | **—** | **40** | **13** | **14** | **—** | **6** |
| Order Anguilliformes | — | — | 4 | — | — | 3 | — | — | — | 6 |
| *Anguilla spp.* | — | — | 4 | — | — | — | — | — | — | 6 |
| *Conger spp.* | — | — | — | — | — | 3 | — | — | — | — |
| Order Beloniformes | — | — | — | — | — | 7 | — | — | — | — |
| Exocoetidae family | — | — | — | — | — | 3 | — | — | — | — |
| Hemiramphidae family | — | — | — | — | — | 3 | — | — | — | — |
| Order Gobiiformes | — | — | — | — | — | 7 | — | — | — | — |
| *Callogobius spp.* | — | — | — | — | — | 7 | — | — | — | — |
| Order Labriformes | — | — | — | — | — | — | 6 | — | — | — |
| *Pseudolabrus spp.* | — | — | — | — | — | — | 6 | — | — | — |
| Order Scombriformes | — | — | — | — | — | 7 | — | — | — | — |
| Scombridae family | — | — | — | — | — | 7 | — | — | — | — |
| *Scomber australisicus* | — | — | — | — | — | 3 | — | — | — | — |
| *Thunnus spp.* | — | — | — | — | — | 3 | — | — | — | — |
| Order Syngnathiformes | — | — | — | — | — | 3 | — | 5 | — | — |
| Mullidae family | — | — | — | — | — | 3 | — | — | — | — |
| Syngnathidae family | — | — | — | — | — | — | — | 5 | — | — |
| *Phyllopteryx taeniolatus* | — | — | — | — | — | — | — | 5 | — | — |
| Order Tetraodontiformes | — | — | — | — | — | 30 | 6 | 10 | — | — |
| Aracanidae family | — | — | — | — | — | 7 | — | — | — | — |
| *Diodon spp.* | — | — | — | — | — | 3 | — | 5 | — | — |
| *Acanthaluteres spp.* | — | — | — | — | — | 23 | — | 5 | — | — |
| *Tetractenos glaber* | — | — | — | — | — | — | 6 | — | — | — |
| Order "undefined" | — | — | — | — | — | 3 | — | — | — | — |
| *Abudefduf spp.* | — | — | — | — | — | 3 | — | — | — | — |
| Order Uranoscopiformes | — | — | — | — | — | 7 | — | — | — | — |
| *Kathetostoma spp.* | — | — | — | — | — | 7 | — | — | — | — |
| ***Amphibia*** | **—** | **—** | **—** | **—** | **—** | **3** | **—** | **14** | **—** | **—** |
| Order Anura | — | — | — | — | — | 3 | — | 10 | — | — |
| Hylidae family |  |  |  |  |  |  |  |  |  |  |
| Limnodynastidae family | — | — | — | — | — | — | — | 5 | — | — |
| ***Reptilia*** | **—** | **33** | **—** | **27** | **35** | **—** | **—** | **19** | **20** | **19** |
| Order Squamata | — | 8 | — | — | 5 | — | — | 14 | — | 19 |
| Elapidae family | — | — | — | — | — | — | — | 5 | — | — |
| Scincidae family | — | 8 | — | — | 5 | — | — | 5 | — | — |
| Order Testudines | — | 25 | — | 27 | 30 | — | — | 5 | 20 | — |
